# Detection of parasitic helminths in cattle from Banda Aceh, Indonesia

**DOI:** 10.14202/vetworld.2019.1175-1179

**Published:** 2019-08-05

**Authors:** Muhammad Hanafiah, Dwinna Aliza, Mahdi Abrar, Fadrial Karmil, Didy Rachmady

**Affiliations:** 1 Parasitology Laboratory, Faculty of Veterinary Medicine, Universitas Syiah Kuala, Banda Aceh, Indonesia; 2 Pathology Laboratory, Faculty of Veterinary Medicine, Universitas Syiah Kuala, Banda Aceh, Indonesia; 3 Laboratory of Microbiology, Faculty of Veterinary Medicine, Universitas Syiah Kuala, Banda Aceh, Indonesia; 4 Laboratory of Clinic, Faculty of Veterinary Medicine, Universitas Syiah Kuala, Banda Aceh, Indonesia; 5 Laboratory of Animal Production, Department Animal Husbandry, Faculty of Agriculture, Universitas Syiah Kuala, Banda Aceh, Indonesia

**Keywords:** Aceh cattle, gastrointestinal parasites, *Oesophagostomum*, *Setaria*

## Abstract

**Aim:**

The objective of this research was to identify the parasite species found in the gastrointestinal tract and pancreas of Aceh cattle slaughtered in a Banda Aceh slaughterhouse using lactophenol and semichon carmine staining.

**Materials and Methods:**

Each sample out of 50 samples of gastrointestinal tract and pancreas from Aceh cattle slaughtered in a Banda Aceh slaughterhouse was separated by organ. Each organ was examined for the presence of worm. Then, the parasitic worms found were subsequently collected and separated based on class and species, followed by staining using lactophenol and semichon carmine. The worms were then identified and their prevalence was determined.

**Results:**

The results showed that three species of parasites were successfully identified, all belonging to the nematode class, namely, *Oesophagostomum radiatum, Oesophagostomum columbianum*, and *Setaria labiatopapillosa* with the prevalence of 12%, 10%, and 6%, respectively. In addition, there was one species of parasite from the trematode class, namely, *Eurytrema pancreaticum* with prevalence of 0.4%.

**Conclusion:**

The nematode class worms, such as *O. radiatum*, *O. columbianum*, and *S. labiatopapillosa*, can be stained by lactophenol, while the trematode class worm such as *E. pancreaticum* can be stained by semichon’s carmine.

## Introduction

In developing countries, the growth and development of health ruminants have not been maximally exploited due to obstacles such as malnutrition, mismanagement, and diseases [1]. Parasitic diseases contribute to the lower rate of animal production in different countries, specifically in tropical and subtropical regions. Ruminants maintained in intensive and extensive systems are very susceptible to various parasitic helminths [2]. Aceh cattle are a crossbreed between local cows (allegedly derived from *Bos sondaicus*) and zebu, derived from India (*Bos indicus*) which the cross bread occurred hundreds of years ago [3]. Bakhtiar *et al*. [4] stated that Aceh cattle genetically have some advantages, for instance in the ability to adapt to disadvantageous environmental conditions such as extreme climate and weather, the ability to reproduce despite poor food conditions, and the ability to withstand several diseases such as gastrointestinal parasites. Gastrointestinal parasites of ruminants may adversely affect their hosts either clinically or economically. The clinical effects which are common in small ruminants are abnormal signs of the dermal, gastrointestinal, and cardiovascular system, while the economic effects result in a smaller genetic potential rate, feed conversion, development, reproduction, and less production of milk or meat. Economic loss for the owners of ruminants most often occurs in morbidity and mortality cases. Understanding the interaction of parasitism, nutrition, and livestock management is the key to managing economic loss [5]. The prevalence of gastrointestinal helminths dairy cattle in the Lampung Province was about 21.60%. This result was 73% lower than the prevalence of helminths in cattle found at Benowo landfill, Surabaya. To solve this problem, integrated gastrointestinal nematode control, consisting of pasture management, rotational grazing, genetic selection, nematicidal fungi, herbal medicines, and anthelmintic treatment, has already been implemented for small ruminants.

Recently, the identification of the worm parasite has mostly focused on the examination of eggs as a basis for worm genus determination, although it is commonly known that many parasites eggs are relatively similar. To further specify worm identification, a staining method using lactophenol and semichon carmine was suggested. The use of the lactophenol and semichon carmine method resulted in the visual transparence of the worm’s body, which clearly displays the morphology of the anterior part, posterior part, and visceral organs of its body. In this way, it is easier to identify whether the species is a nematode or trematode worm. The body of nematode worms are surrounded by a thick cuticle, which prevents the staining solution to absorbed into this layer and the visceral organs as well. Therefore, the lactophenol solution is suggested for use since it promises a higher quality of results. In this method, the worm’s body must be soaked thoroughly in the solution to avoid the worms becoming dry, which would result in the damage of the worm’s body during staining. The soaking time in the solutions, particularly in xylol solution, should not be too long. Due to the thinness of the worm, a long time in the solution causes the color of the worm to become too dark, thus difficult to examine microscopically. [6].

The objective of this research was to identify the helminth parasites in the gastrointestinal tract and pancreas of Aceh cattle slaughtered in Banda Aceh slaughterhouse using lactophenol and semichon carmine staining.

## Materials and Methods

### Ethical approval

All stages of the research were approved by the Veterinary Ethics Committee Faculty of Veterinary Medicine, Universitas Syiah Kuala, Banda Aceh, Indonesia (Approval no. 06/KEPH/VH/2018).

### Sample preparation

For identification of parasite species in gastrointestinal tract and pancreas of Aceh cattle slaughtered in a Banda Aceh slaughterhouse, a total of 50 samples of each organ were collected then separated, according to the organ. Each sample section was examined for the presence of worms. Worms found were separated by class and stained by lactophenol (two parts glycerin; one part crystal phenol (liquid); one part lactic acid; and one part distilled water) and semichon carmine. The research was conducted from February to September 2018.

### Lactophenol staining method

Nematodes were soaked in lactophenol for 24 h until their cuticle turned transparent. Nematodes with a soft outer layer were put into AFA liquid (alcohol, formalin, and acetic acid). Worms were then placed on a glass microscope slide, a drop of lactophenol was placed on the worm, and then both were covered with a cover glass. The slide was examined using a light microscope with 100× magnification.

### Semichon carmine staining method

### Fixation process

Whole trematodes were selected and then pressed between two microscope slides before being bound by a rubber band in order to flatten them. They were submerged in AFA liquid inside a Petri dish for 24 h. (The worm staining process took around 3.5 h. In the case of incomplete processing, the staining product would fail).

### Staining process

White porcelain basins with several pits were used. Worms were released from between the microscope slides and then inserted into a series of four liquids, in this order: distilled water, 30% alcohol, 50% alcohol, and 70% alcohol, for 15 min each. Fifteen drops of 70% alcohol and fifteen drops of semichon carmine liquid were placed in a white porcelain spotting plate depression and stirred by a needle tip. Worms were put into the liquid for 60 min. Afterward, the worms were placed in an alcohol series (70% alcohol, 80% alcohol, and 95% alcohol) for 15 min each, then placed into a mixture of 95% alcohol and two drops of HCl, and placed into absolute alcohol followed by xylol for 15 min each. Worms were then mounted on a microscope slide. The samples were examined under a light microscope with 40× magnification. Worm identification through morphological feature was done according to the standard method developed by Cable [7].

## Results and Discussion

**Figure-1:**
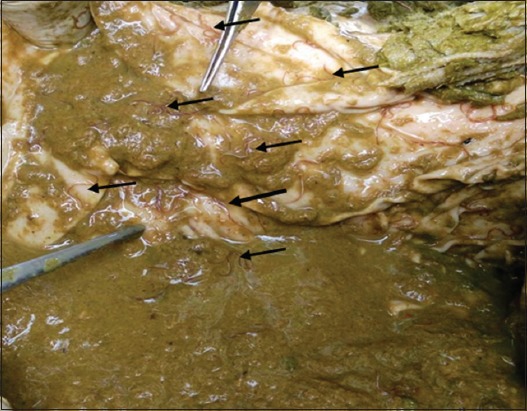
Nematodes found in Aceh cattle gastrointestinal tract (arrows).

The results of worms from 50 samples of gastrointestinal tract and pancreas collected from Aceh cattle (Figure-1) culled in a Banda Aceh slaughterhouse are shown in Table-1, consisting of nematodes and trematodes species.

**Table 1:** The species parasite was found in Aceh cattle slaughtered at a Banda Aceh Slaughterhouse (n=50).

Worm	Class	Predilection	Positive	Negative	Prevalence (%)
*Oesophagostomum radiatum*	Nematode	Large intestine	6	44	12
*Oesophagostomum columbianum*	Nematode	Large intestine	5	45	10
*Setaria labiatopapillosa*	Nematode	Small intestine	3	47	6
*Eurytrema pancreaticum*	Trematode	Pancreas	2	48	4

**Figure-2:**
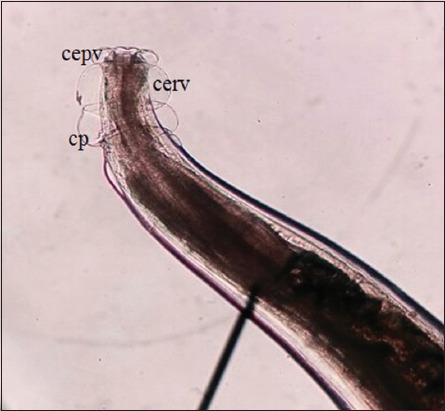
Photomicrograph showing the anterior end of *Oesophagostomum radiatum* nematode found in Aceh cattle 100×. cepv = Cephalic vesicle, cerv = Cervical vesicle, cp = Cervical papillae.

**Figure-3:**
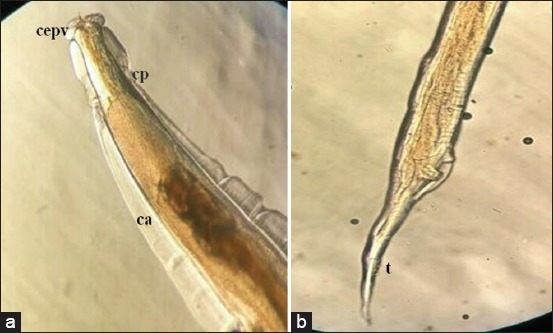
Photomicrograph showing (a) the anterior end of *Oesophagostomum columbianum* nematode found in Aceh cattle 100×. Cepv = Cephalic vesicle, cerv = Cervical vesicle, cp = Cervical papillae, ca = Cervical alae; (b) The anterior end t = tail.

**Figure-4:**
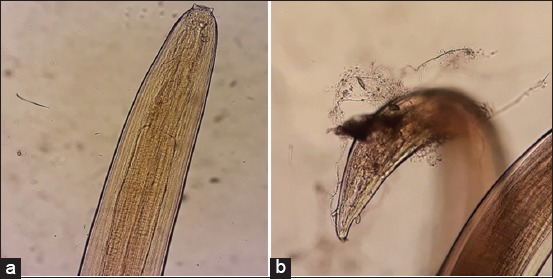
*Setaria labiatopapillosa* nematode in Aceh cattle. (a) Female worms - cephalic end; (b) female worms - caudal end.

As shown in Table-1, the parasite species found in the gastrointestinal tracts of Aceh cattle were *Oesophagostomum radiatum* (Figure-2), *Oesophagostomum columbianum* (Figure-3), *Setaria labiatopapillosa* (Figure-4), and *Eurytrema pancreaticum* with the prevalence of 12%, 10%, 6%, and 4%, respectively. This prevalence is lower compared to research conducted by Hindun *et al*. [8], on dairy cows in Lampung Province, which resulted in the following prevalence: *Haemonchus* spp. 40.6%, *Paramphistomum* spp. 37.5%, *Mecistocirrus* spp. 12.5%, and *Oesophagostomum* spp., *Cooperia* spp., and *Bunostomum* spp. 3.15%.

The difference in nematode prevalence in various regions are influenced by several factors, for instance, infection agent, age, sex, breed, feed, and farming management [9-11]. Variation may be due to change in management practices of different herds and opportunity for grazing.

Tolistiawaty *et al*. [12] stated that herd farming management highly influences parasite infection prevalence. Setiadi *et al*. [13] stated that cattle farming management patterns in Indonesia can be grouped into three categories: Extensive (pasturage), intensive (penned), and semi-intensive combination pattern (traditional). The use of the semi-intensive system by letting the cattle graze for feed (pasture system) and traditional system which are cattle not penned at all will increase the risk of worm infection while cattle that managed intensively, the infection risk can be reduced since feed is given in the pen. The dairy cattle farming system in the Lampung Province is already intensive, whereas Aceh cattle in the Aceh region are mostly managed extensively, without pens and semi-intensively by grazing them during the day and then penning them during the night. This system provides a lot of opportunities for worm larvae in grasses to infect cows in their pastures. This is worsened by the habit of some farmers to release their cattle in the morning.

Other important factors influencing parasite infestation are body weight and breed. Alencar [14] supports the claim that cow breed also influences the presence of the parasite; furthermore, crossbred animals are more resistant to worm infection compared to purebred livestock maintained in tropical conditions. This claim, however, is slightly different from Yusmadi and Jamaliah [4] which stated that genetically, Aceh cattle have the advantage of adaptive ability toward disadvantageous environmental conditions, such as extreme climate and weather, the reproductive ability even with low-quality feed, and survival ability against several diseases, including parasitic diseases. The variation in prevalence depends on the difference in agro-climatic condition and availability of susceptible host [15].

Singh *et a*l. [15] found that the overall prevalence of parasitic infection was significantly higher in females than males. In sheep, a significantly (p < 0.01) higher prevalence was recorded in females (87.38%) as compared to males (72.41%). The influence of sex on the susceptibility of animals to infections could be attributed to genetic predisposition and differential susceptibility to hormonal control. The physiological peculiarities of female animals usually constitute stress factors which reduce their immunity to infections, and for lactating mothers, females can become weak and malnourished, and therefore are more susceptible to the infections [16,17].

Kadarsih [18] added that age heavily influences the gastrointestinal parasite infestation process in young animals. Furthermore, Zulfikar and Razali [19] added that nematodes can survive in the host and cause chronic infection (lasting 1-10 years) and had the ability to avoid the host’s defense system and cause re-infection in adult cattle.

Purwaningsih *et al*. [20] reported that the other factors influencing the distribution of nematode worms between animals are sanitation and pen hygiene. The feces that accumulates in the pens attracts the flies, and is a suitable environment where nematode larvae can flourish. When the skin of cattle makes contact with the dirt, the larvae can infiltrate into host’s body.

*Setaria labiatopapillosa* (*syns., Setaria cervi*) (Figure-4) prevalence in Aceh cattle slaughtered in a Banda Aceh slaughterhouse is 4%. This result is much lower compared to research carried out Bino and D’Souza [21] which found that out of the 500 cattle screened, 187 (37.4%) were found harboring Setaria worms in the peritoneal cavity. Three species of Setaria, namely, Setaria digitata, S. cervi, *and* S. labiatopapillosa were observed in the Bino study. Out of the 187 cattle which were positive for Setaria, 106 (56.8%) had S. digitata, 45 (24.13%) had S. cervi, and 36 (18.96%) had S. labiatopapillosa. Thirty animals (16.04%) were found to be infected by all three *Setaria* spp. The worms were found freely in the peritoneal cavity or attached to the intestines, mesentery, walls of the peritoneum, lungs, liver, heart, urinary bladder, uterus, and fascia. Some worms were found embedded in patches of inflammatory tissue attached to the visceral walls of the pelvic peritoneum.

The average body length and width of S. labiatopapillosa female worms were 150 and 0.60 mm, respectively, whereas that of S. labiatopapillosa male worms were 80 and 0.40 mm, respectively. S. labiatopapillosa females had a prominent peribuccal crown with rectangular lateral lips (Figure-4a), and the tail end had a smooth button with pointed lateral appendages (Figure-4b). The anterior end of *S. labiatopapillosa* is rounded, with a cuticular peribuccal ring bearing two lateral prominences, a notched dorsal, and ventral prominences.

**Figure-5:**
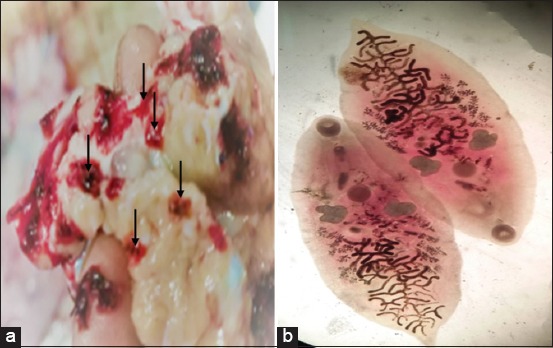
*Eurytrema pancreaticu*m trematode found in Aceh cattle. (a) Worms found in cattle pancreas (arrows); (b) *E. pancreaticum* worm stained with semichon carmine.

The trematode *E. pancreaticum* is a parasite of ruminant pancreatic and bile ducts and also occasionally infects humans, causing eurytremiasis. In spite of it being a common fluke of cattle and sheep in endemic regions, little is known about the genomic resources of the parasite [22]. This research also found *E. pancreaticum* (Figure-5) with a prevalence of 0.4%. Morphologically, *E. pancreaticum* is longer and wider compared to other *Eurytrema* spp. The mouth suction is stronger compared to the abdominal suction. The data of *E. pancreaticum* are also supported by Mirza and Kurniasih [23] who had identified the *Eurytrema* genus from Aceh, Yogyakarta to Makassar, and found three species of *Eurytrema*, which are *E. pancreaticum, E. dajii*, and *Eurytrema* spp. *E. pancreaticum* can only be found in samples originating from Aceh. *E. dajii* can be found on any samples, whereas *Eurytrema* spp. can only be found in samples from Makassar. The result of this research strengthens the theory that *E. pancreaticum* is present in cattle in Aceh.

## Conclusion

Nematode class worms such as *O. radiatum, O. columbianum*, and *Setaria labiatopapillosa* can be stained by lactophenol, while trematode class worms such as *E. pancreaticum* can be stained by semichon carmine.

## Author’s Contributions

MH and DA supervised the overall research work. MA, FK, and DR participated in sampling, made available relevant literature, executed the experiment and analyzed the worms and data. All authors interpreted the data, critically revised the manuscript for important intellectual content and approved the final version.
